# Understanding the Exceptional Properties of Nitroacetamides in Water: A Computational Model Including the Solvent

**DOI:** 10.3390/molecules23123308

**Published:** 2018-12-13

**Authors:** Giovanni La Penna, Fabrizio Machetti

**Affiliations:** 1 Istituto di Chimica dei Composti Organometallici (ICCOM), Consiglio Nazionale delle Ricerche (CNR), via Madonna Del Piano 10, I-50019 Sesto Fiorentino, Firenze, Italy; 2 Istituto di Chimica dei Composti Organometallici (ICCOM), Consiglio Nazionale delle Ricerche (CNR), c/o Dipartimento di Chimica “Ugo Schiff” via Della Lastruccia 13, I-50019 Sesto Fiorentino, Firenze, Italy

**Keywords:** amides, carbanions, C–H acidity, nitro-aci tautomerism, molecular dynamics, density-functional theory

## Abstract

Proton transfer in water involving C–H bonds is a challenge and nitro compounds have been studied for many years as good examples. The effect of substituents on acidity of protons geminal to the nitro group is exploited here with new p$K_{a}$ measurements and electronic structure models, the latter including explicit water environment. Substituents with the amide moiety display an exceptional combination of acidity and solubility in water. In order to find a rationale for the unexpected p$K_{a}$ changes in the (ZZ${}^{\prime }$)NCO- substituents, we measured and modeled the p$K_{a}$ with Z=Z${}^{\prime }$=H and Z=Z${}^{\prime }$=methyl. The dominant contribution to the observed p$K_{a}$ can be understood with advanced computational experiments, where the geminal proton is smoothly moved to the solvent bath. These models, mostly based on density-functional theory (DFT), include the explicit solvent (water) and statistical thermal fluctuations. As a first approximation, the change of p$K_{a}$ can be correlated with the average energy difference between the two tautomeric forms (*aci* and *nitro*, respectively). The contribution of the solvent molecules interacting with the solute to the proton transfer mechanism is made evident.

## 1. Introduction

**Figure 1:**
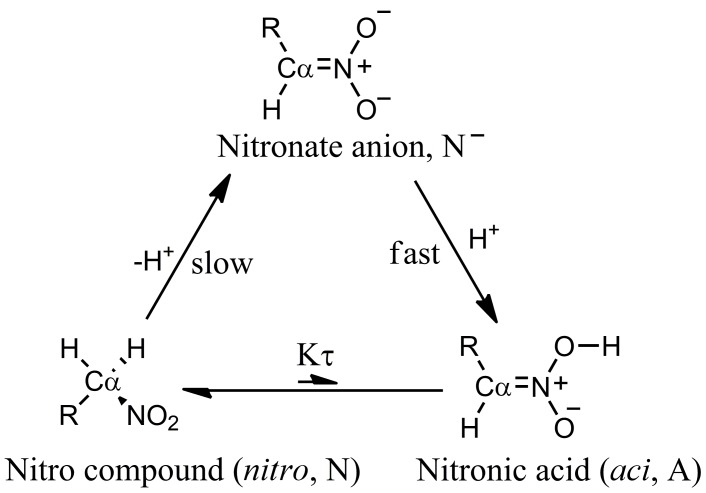
Schematic picture of species involved in the acid-base equilibria of nitro compounds.

Nitro compounds are useful reagents in synthetic organic chemistry [1]. They are precursors of dipoles in 1,3-dipolar cycloaddition [2,3,4,5,6], a source of carbon nucleophiles in conjugated additions [7,8] and nitro aldol (Henry) reaction [9], and a substrate in Nef reaction [10,11]. In all of these reactions, C–H protons geminal to the nitro group are involved. Because of the presence of the nitro group, the above C–H protons show a higher degree of acidity (compound **4**, Table 1) compared with the C–H protons of an aliphatic chain. This feature is due to the ability of the nitro group to stabilize the carbanion in the form of the nitronate anion. The species involved in the nitro compound acidity are depicted in Figure 1 for primary nitro compounds.

An interesting aspect of nitro compounds is their lower proton extraction rate from Cα than that expected from the acidity (Figure 1). This aspect is due to the required conformational rearrangement of the Cα atom (from $sp^{3}$ to $sp^{2}$) to delocalize the negative charge of the carbanion to the nitro group [12]. The p$K_{a}$ of nitronic acid, as it can be derived by kinetic experiments [12], is about 3.5. The issue of the unusual acidity of nitro compounds with labile C–H bonds in a geminal position has been the object of experimental and modeling studies for a long time [12,13,14].

During our work on condensation of nitro compounds with alkenes or alkynes, we became interested in mechanistic aspects of this reaction [6,15,16]. We envisaged that acid-base properties of the substrates could be involved. The acidity of nitro compounds is enhanced by electron withdrawing groups such as esters and ketones in geminal position, resembling carboxylic acid in acid strength (compound **5** vs. compounds **6**–**8**, Table 1) [12]. Intramolecular interactions, including hydrogen bonds, stabilize to different extents the species involved.

**Figure 2:**
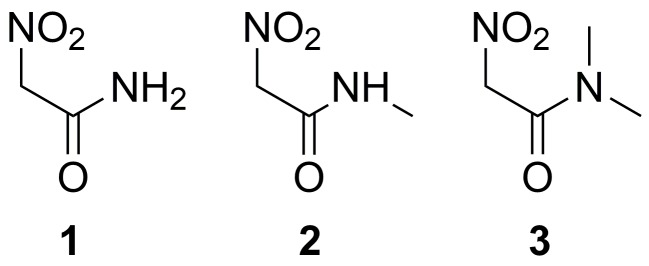
Nitroacetamides studied in this work.

Therefore, in this work, we complete the list of ionization constants for some nitro compounds, including the nitroacetamides **1**–**3** (Figure 2), which are the major focus of our study because of the exceptional combination of acidity and solubility of compound **1**.

Unexpectedly, nitroacetamides **1**–**3** show significant change in p$K_{a}$ values by replacing N-CH3 methyl groups in **3** with protons (compounds **2** and **1**, Table 1). As we show with computational models, those values cannot be easily explained with stabilization factors on nitronate ions. In addition, the prediction of p$K_{a}$ using a popular software [22], available from the SciFinder™ database, does not completely agree with the experimental data (Table 1, last column).

To provide a rationale for the effect of amide derivatives on the acidity of C–H bonds in geminal position to the nitro group, we present in this work an original model where, in addition to electronic and steric intramolecular effects, the role of the water solvent is included. Electronic effects are included using density-functional theory (DFT) with exchange functional described as in the Perdew–Burke–Ernzerhof (PBE) approximation [23], when dynamical methods are used [24,25], or in the Becke three-parameter Lee–Yang–Parr (B3LYP) approximation [26], when static (or single point) calculations are performed. The models, compared to quantum mechanics/molecular mechanics (QM/MM) techniques [27], allow the study of subtle effects due to charge separation during the addressed reaction [28].

It is found that the solvent exerts an essential effect that opens to a new design strategy for further enhancing this important type of acidity.

## 2. Results and Discussion

Following the analysis first reported in Ref. [19], the “apparent” ionization constant $K^{\prime }$ is a function of the ionization constants of the two tautomeric forms, respectively *aci* and *nitro* (see Figure 1):

(1) $$K^{\prime }=\frac{\left[N^{-}\right]\left[H_{3}O^{+}\right]}{\left[A]+[N\right]}.$$

Manipulating the equation above, the apparent $K^{\prime }$ constant can be expressed in terms of the equilibrium constant between the two tautomeric forms $K_{\tau }$:

(2) $$K^{\prime }=\frac{K_{N}}{\left(K_{ø}+1\right)}≃K_{N},$$

where $K_{N}$ is the ionization constant of the nitro form (that is the most stable at room conditions) and $K_{\tau }$ = [A]/[N]. The low ratio between *aci* and *nitro* forms ($K_{\tau }<<1$) at room conditions in water solution prevents the species from showing the larger acidity of the *aci* form compared to the *nitro*. The former is more acidic because the C–H bond is always stronger than the O–H bond. However, the enhanced chemical properties of rare species present with very low statistical weight in the sample are evident in the measured apparent ionization constant. The stronger acidity of the low-weight *aci* form is evident when the proton exchange between the *aci* form and the *nitro* form is frozen or the kinetics of the *aci* deprotonation can be separated by measured kinetic data [12]. Hereafter, we indicate $K^{{}^{\prime }}$ as $K_{a}$.

The prediction of p$K_{a}$ for the compounds displayed in Figure 2 is a challenging task also for empirical methods, the latter still the more accurate [29]. The application of a a popular software [22], available from the SciFinder™ database, does not agree with the experimental data (see Introduction above) and our work aims at explaining the disagreement in terms of atomistic models. Theoretical and computational methods achieved significant advancement, but reliable applications are still problematic when protons are released by C atoms, rare species are transiently involved and subtle effects of solvent, especially water, play a role in the thermodynamics of the proton exchange.

From a microscopic point of view, the contribution of rare acidic forms to the average observed property, that is potentially dominated by low-acidic forms, can be explained if the reactive form is trapped within energy barriers. In this case, the conversion from the rare form to the most stable one is slower than the ionization. The average property, provided by the series of sampled microscopic states, slowly converges with sampling.

Indeed, this effect can be achieved in practice with computational models where the model is constrained towards bound states and cannot escape from one chemical configuration to another. Among these models, the tight-binding method forces the sampling of bound states. In this approximation, the sampling of rare chemical species can last for a long time even if in theory the atoms should rapidly change the valence to reach the most stable configuration. Therefore, despite the many limitations of the tight-binding approximation, it is possible to compare the energy of different bound states, while free energy changes are affected by huge errors. In this case, the average energy can be computed in different samples, each mimicking the metastable equilibrium state of the two different and separated tautomeric forms. Another advantage is the possibility to include explicit water molecules in the modeled sample. In this work, we used the self-consistent charge density-functional tight-binding approximation [30] (DFTB, hereafter).

In order to compare the thermodynamic quantities measured by experiments with results of microscopic models, we make the following assumption in the context of the nitro compounds an object of this study. The larger the statistical weight of the *aci* form, the larger the acidity of the sample. The tight-binding approximation can be then used to describe realistic configurations with significant statistical weight for each of the two tautomeric forms. Once this goal is achieved, the proton transfer between the two forms can be described with more detailed computational experiments still including the contribution of the solvation layer. The latter task is accomplished here by adding an external empirical potential to a density-functional theory (DFT) approximation of electron density coupled with molecular dynamics (MD) simulations.

### 2.1. Tight-Binding Approximation of nitro and aci Forms

In Table 2, the difference in average energy ($\Delta E_{\tau }$) at T=300 K and at the water density of bulk water ($\rho_{0}=1$ g/cm${}^{3}$) between the two tautomeric forms is reported.

These data are compared with the measured ionization free energy change and with the same energy difference computed with an accurate DFT approximation that allows geometry optimization in an implicit model of the water solvent. The final column is the $\Delta G_{0}$ derived from p$K_{a}$ values predicted with an empirical method provided by the SciFinder™ database. According to a comparison between different prediction methods [29], the ACDLabs [22] method is one of the best performing.

The approximate DFTB model of the electronic structure and the low statistics are not expected to provide agreement between the measured free energy changes (column 2) and the computed energy difference between ionized and neutral species (not shown here). However, it can be noticed that the most acidic species (less positive free energy of ionization, column 2) display the lowest difference in energy of the *aci* tautomeric form (column 3). With the exception of **6**, the series of substituents displays the correct order for both $\Delta G^{0}$ and $\Delta E_{\tau }$. This rough correlation indicates that the contribution to the apparent acidity due to substituent R can be ascribed to the increasing statistical weight of the *aci* form, the latter characterized by large acidity.

The ACDLabs empirical prediction, though it is excellent for the compounds that are presumably tabulated (**4**–**8**), fails in predicting the high acidity of compound **1** and the decrease of acidity of **3** with respect to **1**.

Intramolecular interactions have only a partial role in determining the average energy difference between the two tautomeric forms. This is shown by the values of $\Delta E_{\tau }$ energy difference computed with the more accurate DFT method (column 4 in Table 2). The values are larger in absolute value than the corresponding DFTB estimate, even though they follow approximately the same ordering, with the smallest absolute values corresponding to the most acidic compounds. The range displayed for the amide derivatives is due to the choice of different structures as initial configurations for the geometry optimization. For instance, the lowest energy *nitro* structure corresponds to an open extended *all-trans* structure, where there is no interaction between the nitro and the amine group. The *aci* form is, compared to this extended structure, at the highest energy. On the other hand, when the *nitro* compound forms intramolecular interactions that favor a closed structure, the *aci* form is at a lower energy. However, in both cases, the energy difference is larger than when the calculation is performed with a less accurate model, but includes the solvent layer explicitly. Therefore, the inclusion of explicit solvent makes the energy landscape flatter than in the case of a polarizable continuum model for the solvent.

The interplay between intramolecular interactions and interactions with solvent molecules is shown by simulations in the explicit solvent.

**Figure 3:**
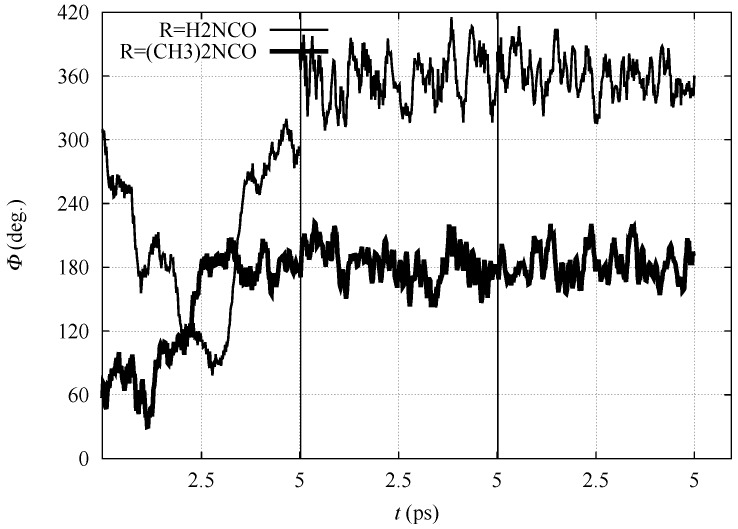
Time evolution of N-Cα-Cβ-Nγ dihedral angle (Φ) with R = H_2_NCO (thin line) and (CH_3_)_2_NCO (thick line) within BO–MD simulations performed with the density-functional tight-binding (DFTB) model. Vertical lines separate the simulation of *nitro* (left), *aci* (middle) and ionized (nitronate) forms (right), respectively.

In Figure 3, the time evolution of the N-Cα-Cβ-Nγ dihedral angle is displayed for all the three simulation stages (*nitro*, *aci* and ionized forms) for **1** and **3**, performed in the DFTB model. The dihedral angle displays for both compounds large fluctuations when in the *nitro* form because of the $sp^{3}$ configuration of Cα. After the displacement of α H to the nitro O atom (in the *aci* form) and then into the bulk water (ionized form), the molecules are sealed into, respectively, E and Z configurations for **1** and **3**. Despite the conformational freezing, keeping the *aci* form in the E configuration, the intramolecular H–N⋯H${}_{N}$-O${}_{N}$-N hydrogen bond is not observed. Also in the ionized form, the O${}_{N}$ atoms of the nitro group strongly interact with water molecules in the solvent (see below).

**Figure 4:**
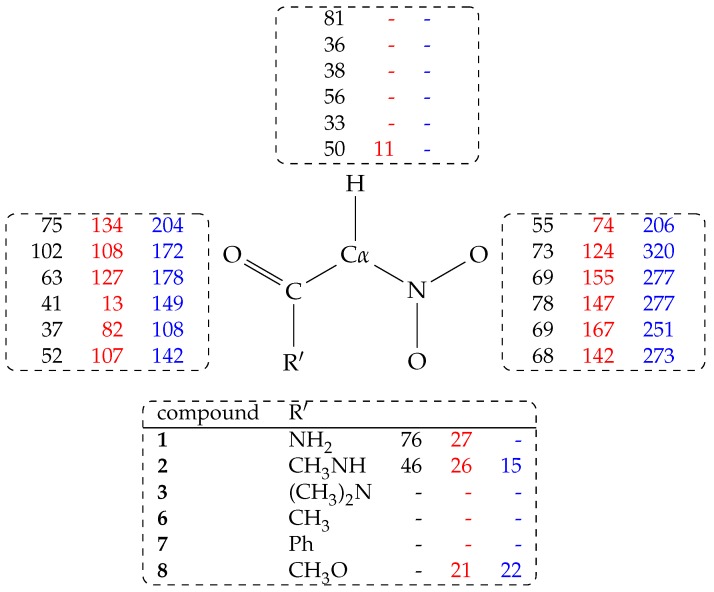
Hydrogen bond population (%) measured as in Table 3 and distributed over atomic groups in *nitro* (black, column 1), *aci* (red, column 2), and nitronate (blue, column 3) species. Lines are in the same order of Table 3. The - symbol indicates no hydrogen bond. Hydrogen bonds are counted for atoms: Cα-H (top); CO (left); NO_2_ (right); R’ (bottom).

Despite the absence of stable intramolecular hydrogen bonds, there are significant intramolecular interactions in certain compounds. For instance, there is a high persistence of the intramolecular interaction between the N–O bond and the amide H atom when R = NH_2_CO (**1**). This interaction keeps the *aci* form sealed in the E conformation, the latter more hindered to water access than the *aci* form of other compounds (see Table 3 and Figure 4 discussed below). The N–O⋯H–N interaction displays an angle smaller than 135${}^{\circ }$, thus being not classified as an hydrogen bond, but rather a strong electrostatic interaction. The proton attached to the nitro group in the *aci* form when R = NH_2_CO never interacts with N and O of the amide group. The latter atom is always *anti* to the nitro group with respect to Cα-C bond. As a consequence of this closed *aci* form, the anion displays always the strong N–O⋯H–N intramolecular electrostatic interaction, while such interaction is not effective in the other substituents.

These observations indicate that the energy of most of the species reported in Table 2 is also strongly modulated by interactions with the water environment, in addition to the electrostatic intramolecular interactions discussed above. The hydrogen bond population of H-bond donor and acceptor groups is reported in Table 3 and in Figure 4.

In this analysis, all the water molecules (213, 210 only when R = PhCO) in the sample are included. From Table 3, it can be observed that the α H atom forms a significant amount of hydrogen bonds with water molecules (Cα-H⋯Ow, with Ow the O atom in water molecules) when Cα is in the *nitro* form. As a term of comparison, when R = CH_3_ (**5**, data not displayed in Table) the probability for Cα-H and Cβ-H hydrogen bonds with water is, respectively, 23 and 22%.

The Cα-H⋯Ow hydrogen bond is lost when the Cα atom is converted into the $sp^{2}$ electronic configuration (*aci* and nitronate forms). The highest population Cα-H⋯Ow hydrogen bond is observed for R = NH_2_CO (**1**) in the *nitro* form (81%, see Figure 4).

The probability of any hydrogen bond with water molecules (both donating and accepting solute hydrogen atoms) reported in Table 3 shows that, for **1** and **2**, and when the solute is neutral, the population of hydrogen bonds has its maximum in the *nitro* form. The probability is still high when the proton is moved to the *aci* form. Finally, when the proton is removed from the solute (the negatively charged nitronate form), the probability of hydrogen bonds with the solvent increases, mainly because of the negatively charged nitro group. Looking at the partition of hydrogen bonds (Figure 4), it can be noticed that substituents with at least one N–H bond are particularly efficient in keeping water molecules structured in the first solvation layer around all the regions of the solute, independently of the tautomeric or protonation form.

The water environment around each molecule has different degrees of basicity and, once it is protonated by the α H extraction, different degrees of acidity towards the solute molecule. To approximately measure acid-base properties of this water environment, α H is extracted from the solute by moving the atom towards the closest water molecule in the solvent bath. In this condition, the water bath (containing a single H_3_O^+^ species) is allowed to give back the proton to the solute. If a short time is provided to the solute, allowing the relaxation of the Cα bond environment, the water bath gives the proton back to other basic groups because, in the tight-binding approximation, the relaxed $sp^{2}$ Cα atom can not form a new C–H bond. Remarkably, in most of the cases, the group that is able to host the proton provided by the water bath is the nitro group, thus forming the solute in the *aci* form. Therefore, this alchemical process mimics a possible pathway for the proton transfer from position Cα to the nitro O atom, as it is mediated by the water layer around the solute. This simple experiment allows a first exploitation of the mechanism by which the solute can better manifest itself as the more acidic *aci* form. Interestingly, in some cases (R = PhCO), the carbonyl oxygen is able to form a transient covalent bond with the proton provided by the water layer.

In Table 4, the times required to transfer the proton from the water layer to one of the oxygen atoms in the solute, producing either the *aci* or the enolic form, is reported.

Each α H extraction to the water layer is performed from a selected configuration displaying an approximately zero or π dihedral angle for αH’-Cα-N–O and a water molecule with Ow within 0.2 nm from α H. It must be noticed that, when these two conditions are not fulfilled, in most of the cases, the α proton is rapidly given back to Cα because there is no efficient relaxation mechanism for the H_3_O^+^ species formed in the water layer.

The formation of the *aci* form from the reaction between the protonated water environment and the negatively charged form of the solute has different lag-times τ displayed in Table 4. In some cases (**1** and **7**), the proton is finally bound by the carbonyl oxygen, forming the enolic isomer of the given species. Only in the case of **3**, the proton goes always back to Cα because of the strong repulsion between the solute and the close by hydronium species formed by the α H extraction. Therefore, these data show that, for **1**, **2** and **7**, the pathway for proton exchange between C–H bond in the solute and a O–H bond in the solvation water layer, followed by the exchange with the O–H bond in the solute, is easily found.

The large chance of formation of enolic forms in the case of **1** and **7** is an indication of the possibility for enolic form as an intermediate in the slow process of Cα deprotonation. A higher probability for enolic form increases the rate for proton release in certain compounds, as observed in the literature [12], because of the $sp^{2}$ pre-organization of Cα. In the DFTB model investigated here, the enolic form appears, in the more hydrophilic nitro compounds analyzed here, as a second acidic form of the nitro compound, in addition to the *aci* form. However, in the DFTB model, the mechanism to obtain the enolic form is mediated by the water molecule close to the α H atom that is extracted.

### 2.2. The α H Extraction from the nitro Tautomer and Insertion into the aci Tautomer

The DFT model of the water solution sample circumvents the limitation of the tight-binding model in oversampling bound states. By using an external force that smoothly extracts one of the α H atom away from the Cα-H bond at room thermal conditions, it is possible to break the C–H bond, keeping the possibility of forming alternative explicit H–O bonds in the first solvent layer of the solute.

**Figure 5:**
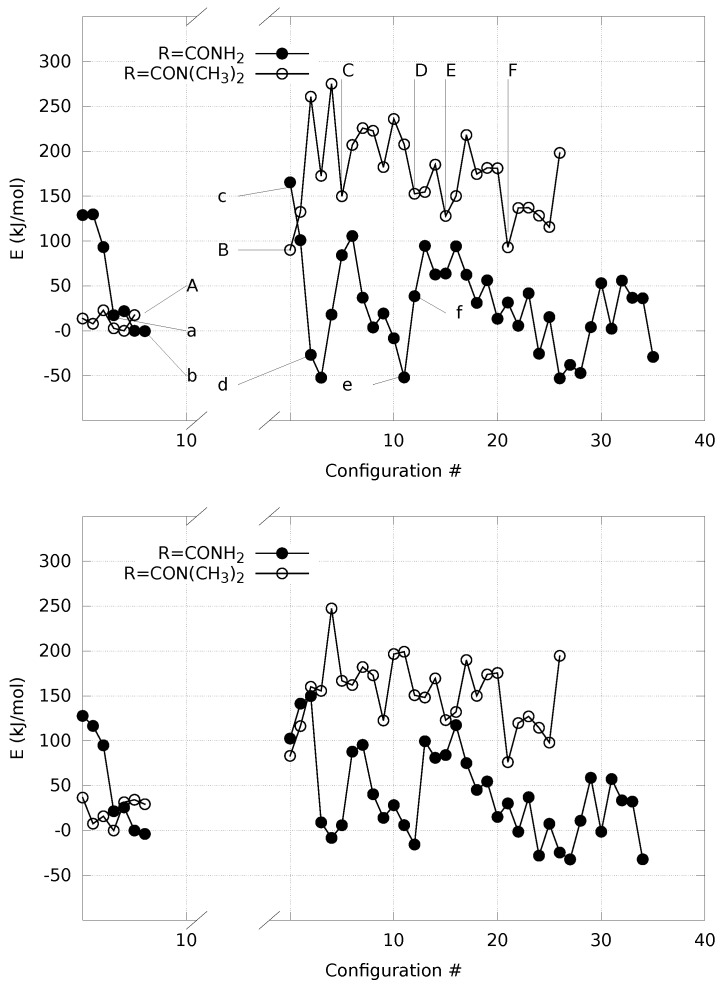
Potential energy with R= H_2_NCO (filled circles) and (CH_3_)_2_NCO (circles) along with the extraction of α H from the bond with Cα atom. Energy is computed for solute within a distance O(water)-solute atoms of 4 Å (see Methods). Energy reference is the lowest energy obtained in the initial *nitro* form for each compound. The gap in the *x*-axis separates *nitro* (left) from nitronate and *aci* (right) species. Arrows in the left panel indicate the configurations displayed in Figure 6 and Figure 7. The calculation is performed with plane-wave basis-set and PBE exchange functional (left) and localized Gaussian basis-set with hybrid B3LYP exchange functional (right panel). Points indicated with a–f and A–F are are displayed in Figure 6 and Figure 7, respectively. See text for details.

The analysis of the change in potential energy along with the α H extraction in the two extreme cases (R = H_2_NCO and R = (CH_3_)_2_NCO) is displayed in Figure 5 (see Methods).

**Figure 6:**
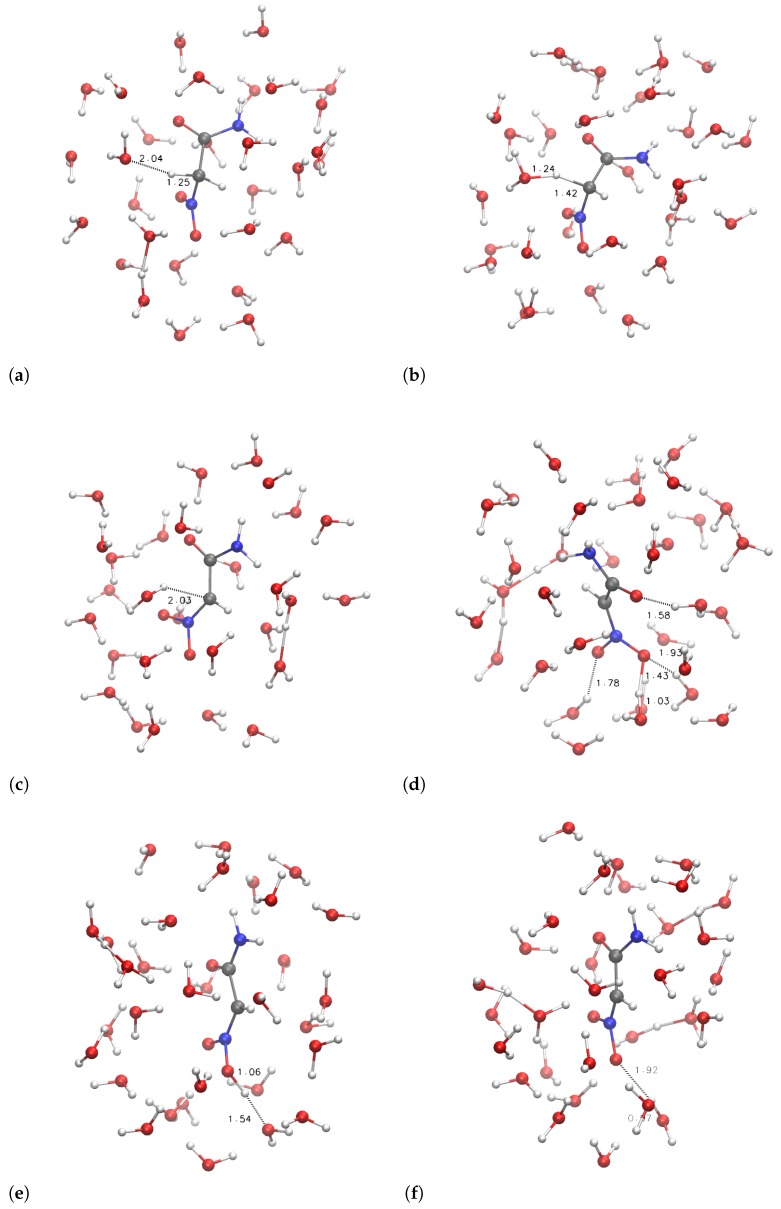
Structures of samples with R = H_2_NCO along with proton extraction (Figure 5). Panels (**a**–**f**) refer, respectively, to points indicated with a–f in Figure 5. C is gray, N is blue, O is red, H is white. Atomic and bond radii are arbitrary. Some relevant distances are displayed. Explicit bonds are drawn when atoms are closer than 1.6 Å. The VMD [31] program is used for all molecular drawings.

**Figure 7:**
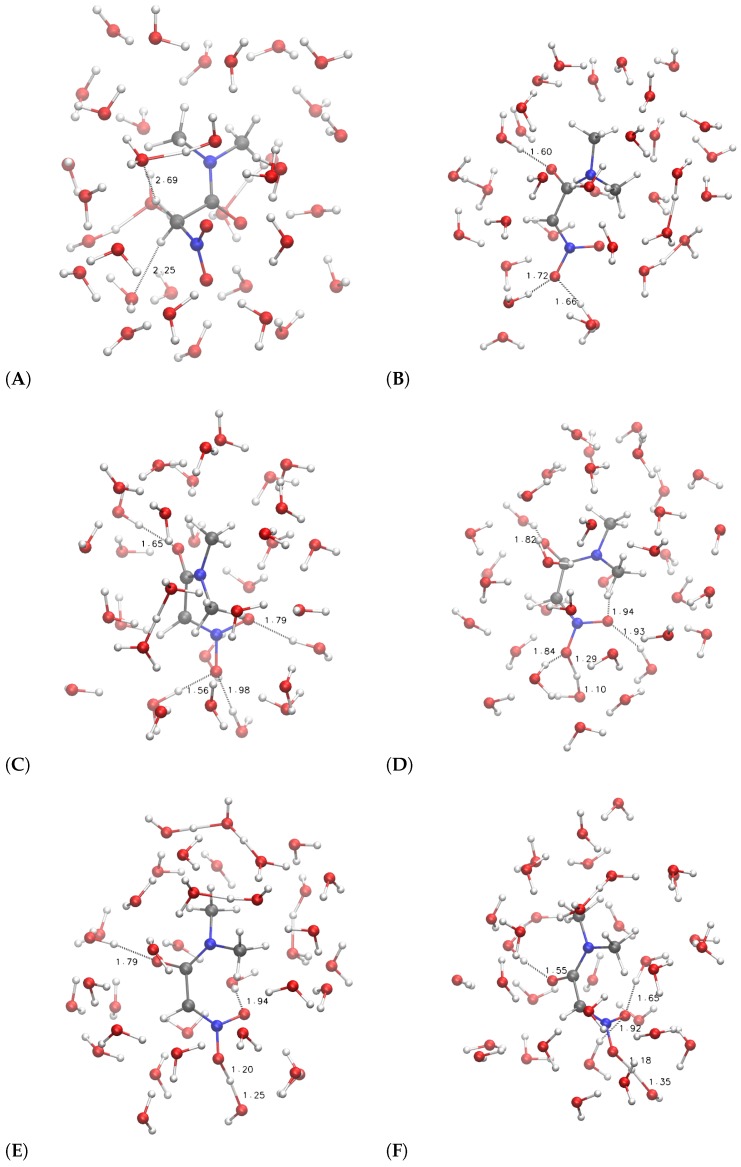
The same as Figure 6 when R = (CH_3_)_2_NCO. Panels (**A**–**F**) refer, respectively, to points indicated with A–F in Figure 5.

The configurations corresponding to some selected points, indicated by letters a–f and A–F, are displayed in Figure 6 and Figure 7, respectively.

It can be noticed that the compact initial structure of **1**, where the electrostatic interaction between the amino and nitro groups is effective, is rapidly lost during equilibration, and extended configurations are sampled in the explicit solvent (Figure 6, panel a). During the application of the external force that drives one of the α H towards the solvent, the more hydrophilic substituent (R = H_2_NCO, filled circles in Figure 5) displays the increase in potential energy due to the exchange of the C–H bond with a O–H bond (Figure 5c). The potential energy is rapidly decreased (∼150 kJ/mol), producing configurations with the proton confined within the solute and a water molecule in the first solvation layer (Figure 6d, the excess proton is on top-right).

On the other hand, the more hydrophobic substituent (R = (CH_3_)_2_NCO, **3**, circles in Figure 5) displays a fast movement of α H to the closest water molecule (2.7 Å compared to 2.0 of **1**), with a similar increase in potential energy compared to **1**. However, the following relaxation of the charge separation (Figure 7C) does not allow a significant decrease in potential energy. The excess proton (that is visible in panel C on top of the carbonyl group) displays a high energy and the movement of the excess proton away from the first solvation layer does not produce a significant decrease in potential energy (Figure 7D). The oscillation of potential energy (panels e–f and E–F of Figure 6 and Figure 7, respectively) does not allow for the hydrophobic substituent (circles in Figure 5) the dissipation of potential energy that is allowed for the more hydrophilic one (filled circles in the same figure).

For both the substituents, the *aci* form is produced during the forced N–O neutralization process (Figure 6 and Figure 7, panels E–F). Nevertheless, the *aci* form is transient and in rapid exchange with anions displaying hydrogen bonds between the nitro group and water molecules in the first solvation layer.

## 3. Materials and Methods

### 3.1. Preparation of Nitroacetamides and Determination of Ionization Constants (Apparent p$K_{a}$)

Nitroacetamide (**1**) and *N*,*N*-dimethylnitroacetamide (**3**) have been obtained, respectively, by aminolysis from ethyl nitroacetate [32,33] and methyl nitroacetate [34], following previously reported procedures.

Ionization constants of nitro compounds **8**, **1**, and **3** were determined in water by potentiometric titration using a glass electrode (method of partial neutralization). The values of pH were determined with CyberScan510 pH meter produced by Eutech Instruments. Compound **8** was used as reference acid and its p*K*${}_{a}$ was determined to reproduce published results [12] using our procedure.

The values of p$K_{a}$ were calculated according to the formula:

(3) $$pK_{a}=\mathrm{pH}+\mathrm{Log}\;\frac{\left[\mathrm{HA}\right]}{\left[A\right]}\;\;,$$

where [HA] is the concentration of non-dissociated nitroacetamide and [A] is the concentration of its salt.

#### 3.1.1. Methyl Nitroacetate (**8**)

A 0.0100 M (59.5 mg in 50 mL) solution of methyl nitroacetate (**8**) (41.0 mL) was titrated with a 0.100 M solution of sodium hydroxyde. Titration data are reported in Table 5.

#### 3.1.2. Nitroacetamide (**1**)

*Run 1*: a 0.0100 M (103.9 mg in 100 mL) solution of nitroacetamide (**1**) (48.0 mL) was titrated with a 0.100 M solution of sodium hydroxyde at T= 23 ${}^{\circ }$C. *Run 2*: the above 0.0100 M solution of nitroacetamide (**1**) (44.0 mL) was titrated with a 0.100 M solution of sodium hydroxyde at T= 21 ${}^{\circ }$C. Titration data are reported in Table 6.

#### 3.1.3. *N*,*N*-Dimethylnitroacetamide (**3**)

*Run 1*: a 0.0100 M (66.1 mg in 50 mL) solution of N,N-Dimethylnitroacetamide (**3**) (43.0 mL) was titrated with a 0.100 M solution of sodium hydroxyde at T= 25 ${}^{\circ }$C. *Run 2*: same as *Run 1* at T= 24 ${}^{\circ }$C. Titration data are reported in Table 7.

### 3.2. Density Functional Tight-Binding (DFTB) Models

The final goal of our models is to investigate the mechanism of the reactions described in Figure 1, within a density-functional theory (DFT) approximation of electrons in a system composed by the solute nitro compound and a sample of solvent water molecules. To accomplish this task, we apply in this work implementations of DFT suited for systems of several hundreds of atoms (see the next subsection below). Before applying time-consuming DFT models to systems composed of several hundreds of atoms, we applied, to the same systems, semi-empirical models that are as close as possible to the final DFT models, in order to minimize effects due to the transition from the semi-empirical to the DFT models. Therefore, we performed molecular dynamics (MD) simulations in the Born–Oppenheimer (BO) approximation and at room conditions (BO–MD, hereafter) within a semi-empirical Hamiltonian describing atomic cores and valence electrons. The Hamiltonian of the system was based on the self-consistent charge density-functional tight-binding approximation [30] (DFTB), because geometrical parameters (like distances and angles) of minimal energy conformations are consistent with accurate DFT calculations for a large set of organic molecules, both isolated and in condensed phases. We used the DFTB+ code [35] for these simulations. The valence electrons of each atom are represented as *s* and *p* orbitals.

We built the solute nitro compounds according to standard geometrical parameters and we merged the resulting solute conformation into a snapshot of the sample of water molecules simulated by MD with the TIP3P interaction potential [36]. This sample is a cubic unit cell with the side of 1.8774 nm containing 216 water molecules, in a configuration extracted by the MD simulated trajectory in the NVT (constant number of particles, volume, and temperature statistical ensemble with T= 300 K and the fixed density of 0.976 g/cm${}^{3}$. The water molecules with the O atom closer than 1.2 Å from any solute atom were discarded. The number of discarded water molecules was in the range from 6 (R = COPh) to 3 (R = CH3CO). As usual, to minimize finite volume effects, periodic boundary conditions are imposed to the system.

The energy of the system was minimized via the conjugate gradient algorithm for 20 steps, in order to reduce the force initially acting on the atoms. Then, the MD simulation in the NVE (constant number of particles, volume, and energy statistical ensemble was performed for 100 steps, starting with velocities extracted from a Gaussian distribution at T= 50 K and with a time-step of 1 fs. During this stage, the temperature never reached values larger than 50 K, indicating the absence of close contacts between atoms. The velocity-verlet algorithm was used to integrate the equations of motion [37]. The MD simulation in the NVT statistical ensemble was then performed continuing the trajectory by using the Nosé–Hoover thermostat [38] at T= 150 K for 1000 steps, followed by 5000 steps (5 ps) at T= 300 K. A unique effective mass corresponding to a coupling constant of 10 THz was used for the thermostat. The second half of the simulation at T= 300 K (2.5 ps) was used for analysis, sampling configurations every 20 fs.

To account for temperature oscillations affecting energy values, the total energy *H* was corrected for the thermal contribution of $N_{deg}RT\left(t\right)/2$, with $N_{deg}=3N_{at}-6$, $N_{at}$ the number of atoms in the simulated cell, and T(t) the actual temperature measured in the system at time *t*. Therefore, the corrected total energy $H^{\prime }=H-N_{deg}RT\left(t\right)/2$ was used for computing the average total energy $E={\langle H^{\prime }\rangle }_{T}$ of each simulated system.

### 3.3. Density Functional Theory (DFT) Models

Car–Parrinello molecular dynamics (CP–MD) simulations [24,25] were performed for models with R = H_2_N, (CH_3_)HN and (CH_3_)_2_N, starting from the final atomic positions and velocities obtained with the corresponding DFTB model at *T* = 300 K. The parallel version of the Quantum-Espresso package [39], which incorporates Vanderbilt ultra-soft pseudopotentials [40] and the PBE exchange-correlation functional [23], was used in all CP–MD simulations. Electronic wave functions were expanded in plane waves up to an energy cutoff of 25 Ry, while a 250 Ry cutoff was used for the expansion of the augmented charge density in the proximity of the atoms, as required in the ultra-soft pseudopotential scheme. As in the DFTB calculations, periodic boundary conditions were applied in the three directions of space. All calculations were performed under spin-restricted conditions, i.e., with two-electrons effective Kohn–Sham orbitals. The electronic ground state was first calculated using 10 steps of conjugate gradient minimization performed on the dynamic variables representing electrons. Then, the system evolution was followed with CP–MD, using as initial velocities those obtained with the final DFTB configuration. As in the DFTB BO–MD, we used the velocity-Verlet algorithm for integrating the equations of motion, with a time step of 0.121 fs. Empirical dispersive corrections of energy and forces [41,42] were included in the CP–MD simulations to correct for the overestimate of atomic repulsion by the DFT approximation.

We performed CP–MD simulations in the NVT statistical ensemble, with temperature held fixed by a Nosé–Hoover thermostat [38]. The systems were equilibrated for 726 fs (6000 time-steps). After this stage, the simulation was continued in the NPT statistical ensemble. In order to further reduce the overestimate of repulsive forces, possibly producing an unrealistic empty space between solutes and solvent water molecules, a short simulation stage at a pressure slightly larger than room conditions can better settle the water layer around the solute. We performed this step with a short simulation (6000 time-steps) with the same thermostat used in the NVT ensemble and a barostat at P= 10 bar [43] with an effective mass of 3/4 $M\pi^{2}$, *M* the total mass of the simulation cell. The cell side oscillates around 1.72 nm in all of the simulations. At the end of this NPT stage, we started manipulating the α H atoms with external pulling forces (see below), still in the NPT statistical ensemble. At the end of the H α extraction from Cα, the sample volume was kept fixed (NVT ensemble).

### 3.4. Pulling αH in DFT Models

We performed pulling experiments in order to explore possible pathways for the mechanism of Cα-H bond breaking, together with the formation of O–H bonds in the water layer around the solute. After extraction of α H from the C–H bond, the O${}_{N}$-H bond formation (with O${}_{N}$ indicating the O atom in the nitro group) was forced in order to achieve the *aci* form of the nitro compound (see Figure 1). To accomplish this pathway, we first applied an external mechanical force on the atoms involved in the Cα-H bonds. When the extraction of α H into the water sample was achieved, we applied a similar external force to the H atoms in the water sample, potentially binding O${}_{N}$ atoms of the nitro group. With the first pulling experiment, we obtain the nitronate anion from the nitro compound in the *nitro* form; with the second experiment, we obtain the *aci* form of the nitro compound starting from the nitronate anion in contact with a protonated sample of water.

As for the first pulling experiment, we defined a collective variable as the Cα coordination number CN according to the equation below [44]:

(4) $$\begin{array}{ccc} CN & = & \sum_{i,j}s_{i,j},\\ s_{i,j} & = & 1\;\;\;\mathrm{if}\;r_{i,j}\leq 0,\\ s_{i,j} & = & \frac{1-{\left(\frac{r_{i,j}}{\sigma }\right)}^{6}}{1-{\left(\frac{r_{i,j}}{\sigma }\right)}^{12}}\;\;\;\mathrm{if}\;r_{i,j}>0,\\ r_{i,j} & = & |r_{i}-r_{j}|-d_{0}\;, \end{array}$$

where the index *j* runs over the α H (two) atoms of the solute and *i* indicates Cα. The actual value of CN can be therefore manipulated by defining an external force as the derivative of an external harmonic potential $U_{e}=\frac{k}{2}{\left(CN-CN_{0}\left(t\right)\right)}^{2}$. By progressively decreasing $CN_{0}$ with the simulation time *t*, we allow the smooth release (when the target $CN_{0}$ value becomes lower than the actual value of CN) of one of the two α H atoms. With this procedure and due to the presence of the explicit water molecules in the model, the C–H bond is broken and, when available, a new O–H bond is formed in the water layer around the solute. The parameter $d_{0}$ in Equation (4) was set to 1.1 Å, while σ was 0.2 Å for the first 363 fs and was increased to 0.5 Å for the following 363 fs. The parameter *k* was set to 1255 kcal/mol in all experiments. The value of $CN_{0}$ is moved from 2 to 1 at the rate of 1 CN value in 2000 CP–MD steps. The pulling of α H was performed in 726 fs after the equilibration.

As for the second pulling experiment, the index *i* runs over the O${}_{N}$ atoms of the nitro group, while *j* runs over all the H atoms not bound to the solute. The $d_{0}$ parameter was 1.03 Å, the latter the equilibrium distance for O${}_{N}$-H${}_{N}$ measured by DFTB simulations. The $CN_{0}$ parameter was increased from zero to one, at the same speed and after the same equilibration time of the α H pulling experiment.

### 3.5. Analysis

We computed a mean-field energy for selected configurations along the pathways sampled with CN manipulations. This calculation is required to correct the energy for contributions due to the periodic boundary conditions and to eliminate thermal fluctuations due to the presence of bulk water around the system of interest (the solute and its hydration layer). From each simulated configuration in the trajectories, we extracted the solute atoms and the water molecules with O atom within 4 Å from any solute atom. For R = H_2_NCO, the number of extracted water molecules is in the range 25–36. For R = (CH_3_)_2_NCO, the same extraction provides a number of water molecules in the range 28–40 because of the larger size of the solute.

When the α H atom becomes farther than 1.6 Å from any solute atom, then α H is not assumed as part of the solute. Thus, α H becomes part of the solvation layer. When the distance between α H and any water molecule in the layer becomes larger than 1.4 Å, then α H becomes part of the bulk solvent and its energy is estimated according to experimental solvation energy of the proton at room conditions [45]. The number of water molecules in the layer changes from one extracted configuration to another. Therefore, the energy contribution due to the addition or deletion of a number *x* of water molecules in the layer is computed according to the estimated energy of isolated water molecules and the cohesion energy per water molecule in the layer (see below). These quantities are computed within the same approximations used for the CP–MD trajectories, except as for the following. In the case of energy calculations, the size of the super-cell was chosen as 2.1 nm, i.e., slightly larger than in the CP–MD simulations (1.8774 nm), to achieve better accuracy in total energy. The wavefunction and density energy cut-off were 30 and 300 Ry, respectively. The Makov–Payne correction [46], accounting for the energy contribution of collecting the charge in the given periodic super-cell, was always included in the reported energies. The water environment, i.e., the bulk water around each solvated solute, was modeled as a uniform dielectric medium with relative permittivity of 78.3 (pure water at room conditions). In these calculations, a self-consistent DFT approach based on plane-waves representation of effective monoelectronic (Kohn–Sham) states was used in place of the dynamical extended Lagrangian method used in CP–MD simulations. We used the implicit solvation scheme implemented in the Quantum Espresso code [47]. The energy tolerance for energy change was 0.01 Ry. All the calculations reported in this work are performed with the contribution of plane-waves with K= 0 in the super-cell lattice described by the periodic boundary conditions used, i.e., in the Γ-point approximation of solid state electron density. Since for water layers the energy minimum cannot be achieved, we performed 30 relaxation steps in the conditions reported above. This number of steps has been found as sufficient to relax most of the vibrational stress in the system.

In order to compare the energy of systems composed of different number of atoms, we used the approximation described below. We first calculated the energy of a single water molecule merged in the dielectric, $E_{w}$, using the same computational conditions of the solvated system (see above). Indicating the different species as in Figure 1, the following equations describe the reaction indicated in the left portion of Figure 1, but including the water layer:

(5) $$N\left[nH2O\right]\to N^{-}\left[\left(n-x\right)H2O\right]+H^{+}+xH2O\;\;.$$

Here, *n* indicates the water molecules in the solvation layer of the N solute, while n−x the number of water molecules in the layer when the nitronate anion N^−^ is formed. The energy change due to the addition or deletion of *x* water molecules (*x* can be a negative number) to the solvation layer is determined by calculating in one single conformation of R = CONH2 the energy change to increase the size of the layer from 16 to 30 water molecules. The cohesion energy $E_{c}$ of a single water molecule to the layer is therefore approximated as:

(6) $$E_{c}=\frac{1}{14}\;\left[E\left(n=30\right)-E\left(n=16\right)+14\;E_{w}\right]\;\;,$$

with $E_{w}$ the energy of the isolated water molecule (see above). We computed this value for a single relaxed configuration of the species R = H2NCO in the *nitro* form. Within this approximation, $E_{c}=$−26.0334 kJ/mol.

Finally, the value of −1107 kJ/mol was used for the solvation energy of H_3_O^+^ [45]. No entropic contribution was taken into account in the calculations reported here, except for the empirical value used for *E*(H_3_O^+^).

The final configurations obtained with the DFTB simulations in the *nitro* and *aci* forms were optimized in an implicit model for water with more accurate DFT approximations. These calculations were performed with Gaussian 16 package [48], using the B3LYP [26] hybrid approximation for the exchange functional and with the 6-31++G(d) basis-set. The PCM method [49] for the implicit water solvation was used. All geometries were optimized according to default “optimization” criteria (Gaussian 16 manual [50]).

## 4. Conclusions

The measurement of apparent ionization constant (p$K_{a}$) for a series of substituted nitromethanes, including the amide moiety (compounds **1**–**3** in Figure 2), shows the strong effect of hydrophobic and bulky sidechains on the Cα acidity. Models including the water molecules interacting with the solute allow for comparing the contribution of intramolecular interactions with that of interactions with structured water layers. This can answer the question about which of these contributions is more efficient to enhance the acidity of the geminal C–H bond.

In this work, we address the inclusion of explicit water molecules in modeling thermodynamic data for this important deprotonation reaction, involving a C–H bond. The reported models, despite the different approximations in the description of ground-state electron density, allow for listing the above observations:

1. The experimental p$K_{a}$ values approximately follow the statistical weight of the *aci* (more acidic) form as a reactant, with the weight measured by the energy of the *aci* form with respect to the low-energy *nitro* form (Table 2).
2. The extraction of α H from the Cα-H bond does not occur necessarily when the molecule populates a closed configuration where the ionized form is stabilized by intramolecular hydrogen-bonds. The work required to extract α H is related to the availability of water molecules near the solute, rather than on the internal structure of the solute itself.
3. The hydronium species (H_3_O^+^) formed in the water solvent is different depending on the nitronate species. When the solute is more hydrophilic (Z=Z${}^{\prime }$=H), the presence of a hydronium close to the solute decreases the potential energy. On the other hand, when the solute is more hydrophobic (Z=Z${}^{\prime }$=CH_3_), a hydronium species close to the solute does not decrease the energy compared to a hydronium species completely separated by the solute.

These observations indicate that the nature of the R substituent, enhancing the acidity of Cα-H, should be hydrophilic in order to increase the probability of persistent hydronium species close to the solute. Intramolecular hydrogen bonds and electrostatic interactions enhancing the population of closed configurations do not appear as requirements for the proton release by the C–H bond. The presence of the amide moiety as a substituent in the geminal position to the nitro group greatly enhances the Cα-H acidity, provided the amide substituent is hydrophilic. The further modification of the amide moiety will be the subject of further studies.

## Figures and Tables

**Table 1:**
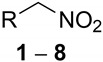
Apparent ionization constants of primary nitro compounds.

Compound	R	p$K_{a}{\;}^{a}$	p$K_{a}{\;}^{b}$
**1**	H_2_NCO	**5.39**; 5.18 [17]	6.20
**2**	(CH_3_)HNCO	5.46 [16]	6.75
**3**	(CH_3_)_2_NCO	**7.23**	5.99
**4**	H	10.7 [18]	10.2
**5**	CH_3_	8.57 [19]	8.49
**6**	CH_3_CO	5.10 [20]	5.40
**7**	PhCO	5.19 [21]	5.37
**8**	CH_3_OCO	**5.70**; 5.68 [16]; 5.56 [12]	5.73

^a^ Data reported in the literature and measured (boldface) in this work; ^b^ Data obtained by ACDLabs available from SciFinder™.

**Table 2:** Comparison between $\Delta G^{0}$ (T=298 K, P=1 bar) for ionization of species RCH_2_NO_2_ soluble in water.

Compound	$\Delta G^{0}$	$\Delta E_{\tau }$ (DFTB)	$\Delta E_{\tau }$ (DFT)	$\Delta G^{0}$
**1**	30.75; 29.55	−13	−53.85/−55.70	35.37
**2**	31.15	−20	−65.16/−73.74	38.51
**3**	**41.25**	−75	−61.54/−66.73	34.17
**4**	61.04	−59	−90.62	58.19
**5**	48.89	−46	−60.32	48.89
**6**	29.10	−24	−56.44	30.81
**7**	29.61	−6	−58.53	30.64
**8**	**32.30**; 32.40; 31.71	−4	−60.14	32.69

Column 2 is derived from p$K_{a}$ in Table 1 (boldface values are obtained in this work). Columns 3–4 ($\Delta E_{\tau }$) are the difference in average energy between *nitro* and *aci* tautomers for the same species. Column 3 is computed in the explicit solvent DFTB model; column 4 is computed in the mean-field solvent DFT model (see Methods for details). Column 5 are values computed from p$K_{a}$ obtained with SciFinder™ (Table 1). All energy values are in kJ/mol. DFTB averages are computed at T=300 K and with water bulk density at T=300 K and P=1 bar. In these conditions, root-mean square error on DFTB energy is in the range 105–140 kJ/mol.

**Table 3:** Hydrogen bond population (%) for all H-bond donor and acceptor groups in the investigated compounds (residue R, see Table 1).

Compound	R	*nitro*	*aci*	*anion*
**1**	H_2_NCO	287	235	410
**2**	(CH_3_)HNCO	257	258	557
**3**	(CH_3_)_2_NCO	170	282	455
**6**	CH_3_CO	175	160	426
**7**	PhCO	139	249	359
**8**	CH_3_OCO	170	281	437

Hydrogen bond is counted when the distance X⋯Y (X donor, Y acceptor) is within 0.3 nm and the X-H⋯Y angle is between 135 and 180${}^{\circ }$. Percentage is obtained as the sum of occurrence of hydrogen bonds over all the water molecules in the sample, the two O–H bonds in water (when donor), acceptor atoms or X-H donating bond of solute, and the configurations collected at T=300 K (250 configurations within the simulation time t=5 ps), finally divided for the number of configurations. Therefore, percentage can be higher than 100.

**Table 4:** Times (τ) required to transfer the excess proton in the water layer, due to α H extraction from Cα, to O atoms either in the nitro group or in the carbonyl group (the latter case indicated with an asterisk).

R	τ (ps)
**1**	0.28 (*)
**2**	1.92
**3**	-
**6**	>5
**7**	0.14 (*)
**8**	>5

**Table 5:** Calculations of the acidity constants of **8** at T= 23 ${}^{\circ }$C (Table 1), according to the results of one titration. p$K_{a}=$ 5.70 as the arithmetic mean of all 12 values in the set.

NaOH (mL)	Log [HA]/[A]	pH	p$K_{a}$
0.3	1.10	4.59	5.69
0.6	0.766	4.88	5.65
0.9	0.551	5.12	5.67
1.2	0.383	5.3	5.68
1.5	0.239	5.42	5.66
1.8	0.106	5.59	5.70
2.1	−0.0212	5.72	5.70
2.4	−0.150	5.84	5.69
2.7	−0.285	5.97	5.68
3.0	−0.436	6.16	5.72
3.3	−0.615	6.33	5.71
3.6	−0.857	6.68	5.82

**Table 6:** Calculations of the acidity constants of **1** (Table 1), according to the results of two titrations. p$K_{a}=$ 5.39 and 5.38 as the arithmetic mean of all eight values in the set *Run 1* and *Run 2*, respectively.

NaOH (mL)	*Run 1*			*Run 2*		
	Log [HA]/[A]	pH	p$K_{a}$	Log [HA]/[A]	pH	p$K_{a}$
0.5	0.934	4.36	5.29	0.892	4.42	5.31
1.0	0.580	4.72	5.30	0.531	4.77	5.30
1.5	0.342	4.96	5.30	0.286	5.02	5.31
2.0	0.146	5.17	5.32	0.0792	5.26	5.34
2.5	−0.0362	5.37	5.33	−0.119	5.48	5.36
3.0	−0.222	5.60	5.38	−0.331	5.75	5.42
3.5	−0.430	5.91	5.48	−0.590	6.21	5.62
4.0	−0.699	6.42	5.72	-	-	-

**Table 7:** Calculations of the acidity constants of **3** (Table 1), according to the results of two titrations. p$K_{a}=$ 7.25 and 7.23 as the arithmetic mean of all 8 values in the set *Run 1* and of 13 values in set *Run 2*, respectively.

**NaOH (mL)**	*Run 1*			NaOH (mL)	*Run 2*		
	Log [HA]/[A]	pH	p$K_{a}$		Log [HA]/[A]	pH	p$K_{a}$
0.5	0.881	6.32	7.20	0.3	1.12	6.11	7.23
1.0	0.519	6.74	7.26	0.6	0.790	6.47	7.26
1.5	0.271	6.99	7.26	0.9	0.577	6.69	7.27
2.0	0.0607	7.2	7.26	1.2	0.412	6.86	7.27
2.6	−0.184	7.41	7.23	1.5	0.271	6.97	7.24
3.0	−0.363	7.62	7.26	1.8	0.143	7.09	7.23
3.5	−0.641	7.91	7.27	2.1	0.0202	7.22	7.24
4.0	−1.12	8.41	7.29	2.4	−0.101	7.34	7.24
-	-	-	-	2.7	−0.227	7.45	7.22
-	-	-	-	3.0	−0.363	7.58	7.22
-	-	-	-	3.3	−0.519	7.73	7.21
-	-	-	-	3.6	−0.711	7.9	7.19
-	-	-	-	3.9	−0.989	8.16	7.17

## Abbreviations

The following abbreviations are used in this manuscript:

DFT	density-functional theory
DFTB	density-functional tight-binding
MD	molecular dynamics
BO	Born–Oppenheimer
B3LYP	Becke three-parameter Lee–Yang–Parr exchange-correlation functional
PBE	Perdew–Burke–Ernzerhof exchange-correlation functional
PCM	polarizable continuum model
